# The complete chloroplast genome of ornamental plant *Ruellia simplex* C.Wright (Acanthaceae)

**DOI:** 10.1080/23802359.2021.1935347

**Published:** 2021-06-17

**Authors:** Feng Siyi, ZhiJun Liu, Tang Hui

**Affiliations:** College of Forestry and Landscape Architecture, South China Agricultural University, Guangzhou City, P.R. China

**Keywords:** *Ruellia simplex*, chloroplast genome, Illumina sequencing, phylogenetics

## Abstract

*Ruellia simplex* C.Wright is a perennial plant of the Acanthaceae, which has significant ornamental value. Because of its strong adaptability, it is widely planted in Chinese rural areas. Based on sequencing data from Illumina, the first complete chloroplast (cp) genome of *Ruellia simplex* C.Wright is reported in this paper. This cp genome was 143,016bp in length, including a large single-copy region (LSC) of 91,857bp, a small single-copy (SSC) of 17,591bp and two inverted repeat regions (IRs) of 16,784bp. It contained 128 genes, 35 transfer RNA genes, 8 ribosomal RNA genes, with an overall GC content of 38.41%. Additionally, the phylogenetic analysis showed that *Ruellia simplex* is closely related to *Strobilanthes cusia* (NC_037485)*, Strobilanthes bantonensis* (MT576695) and *Echinacanthus attenuatus* (NC_039762). The results of this study provide valuable information for the continued study of its species evolution, genetic engineering and germplasm resource utilization.

*Ruellia simplex* C.Wright is native to Central and South America and was introduced into China as an ornamental plant. It is mainly distributed in Guangdong, Guangxi, Hainan, Yunnan provinces, China. It is a perennial plant of the Acanthaceae. Due to its elegant color, beautiful flower and two types of high and low shape, its landscape uses are various, including in flower borders, flower beds, road greening, rock garden planting, potted landscapes and so on. It also has strong adaptability to drought, barren and saline-alkali soil resistance, and especially high-temperature resistance. In China, it makes up for a shortage of flowering plants in mid-summer, and it has become an variety widely used in landscape architecture. A chloroplast is a vital organelle for photosynthesis in plants, and the chloroplast genome can provide information for species evolution research, genetic engineering research and germplasm resource utilization. But chloroplast genome study of *Ruellia simplex* is limited at present, so this paper analyzes its complete chloroplast genome based on Illumina sequencing data. The results provide essential genetic information for the further study of its photosynthetic regulation, plant resistance, species identification and genetic relationships. Besides, it lays a foundation for conducting further chloroplast genetic engineering research.

Fresh leaves of *Ruellia simplex* were collected from Qingtian Village, Shunde District, Foshan City, Guangdong Province, China (113°8′22″ E, 22°48′45″ N), for total genomic DNA extraction. A specimen was deposited at Herbarium of South China Agricultural University (Zheng Ming-Xuan, zhengmx@scau.edu.cn) under the voucher number 32202(CANT). High-throughput DNA sequencing was conducted on the Illumina Novaseq6000 Sequencing System, and the sequencing strategy was PE150 (Pair-End 150) with data volume of no less than 2GB. The software SPAdes v. 3.5.0 (Lapidus et al. 2014) was used to connect the chloroplast genome sequence, and then the CPGAVAS (Liu et al. 2012) and the ORF Finder were used to annotate the chloroplast genome. Next, ARWEN (Laslett and Canbäck 2008) was used to annotate tRNA and the results were verified again by using tRNAscan-SE 2.0 (Lowe and Chan 2016).

The complete chloroplast genome of *Ruellia simplex* (GenBank accession MW697905) was 143,016bp in length, containing a large single copy-region (LSC) of 91,857bp, a small single copy-region (SSC) of 17,591bp, a pair of inverted repeat regions (IRs) of 16,784bp, with an overall GC content of 38.41%. This study annotated 128 genes. The proportion of genes was 58.60%, including 85 protein-coding genes, 35 tRNA genes, 2 rrn16 genes, 2 rrn23 genes, 2 rrn4.5 genes and 2 rrn5 genes. A physical map of the *Ruellia simplex* chloroplast genome was constructed.

To determine the developmental situation of *Ruellia simplex* within Acanthaceae in NCBI databases, we retrieved completions of chloroplast genome sequencing on Acanthaceae using ‘Acanthaceae’ and ‘chloroplast genome’ as the keywords. The results showed that the chloroplast genome sequencings of another 23 species within Acanthaceae have already been completed. A neighbor-joining phylogenetic tree was constructed based on the chloroplast genome sequences of 25 species with the program MEGA6 (Molecular Evolutionary Genetics Analysis) (Tamura et al. 2013). The results illustrated that *Ruellia simplex* is closely related to *Strobilanthes cusia* (NC_037485)*, Strobilanthes bantonensis* (MT576695) and *Echinacanthus attenuatus* (NC_039762) (Figure 1).

**Figure 1.**
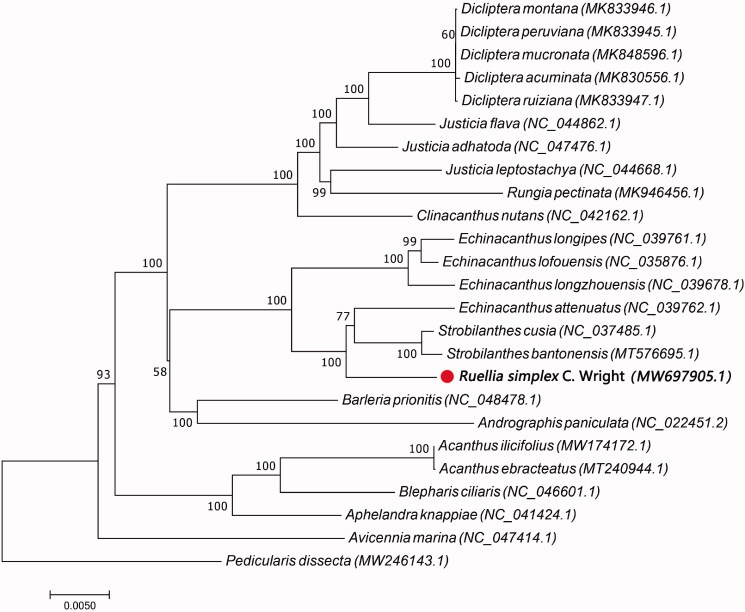
A Maximum parsimony (MP) neighbor-joining phylogenetic tree was constructed using chloroplast genome sequences of 25 species within the Acanthaceae family, and the Orobanchaceae family as outgroup. *Ruellia simplex is marked* with a red circle.

## Data Availability

The genome sequence data that support the findings of this study are openly available in GenBank of NCBI at [https://www.ncbi.nlm.nih.gov] (https://www.ncbi.nlm.nih.gov/) under the accession no.MW697905. The associated BioProject, SRA, and Bio-Sample numbers are PRJNA727707, SRR14454231, and SAMN19030483, respectively.
